# Neuroinvasion of α-Synuclein Prionoids after Intraperitoneal and Intraglossal Inoculation

**DOI:** 10.1128/JVI.01399-16

**Published:** 2016-09-29

**Authors:** Sara Breid, Maria E. Bernis, Julius T. Babila, Maria C. Garza, Holger Wille, Gültekin Tamgüney

**Affiliations:** a German Center for Neurodegenerative Diseases (DZNE), Bonn, Germany; b Centre for Prions and Protein Folding Diseases & Department of Biochemistry, University of Alberta, Edmonton, Alberta, Canada; Rocky Mountain Laboratories

## Abstract

α-Synuclein is a soluble, cellular protein that in a number of neurodegenerative diseases, including Parkinson's disease and multiple system atrophy, forms pathological deposits of protein aggregates. Because misfolded α-synuclein has some characteristics that resemble those of prions, we investigated its potential to induce disease after intraperitoneal or intraglossal challenge injection into bigenic Tg(M83^+/−^:*Gfap*-luc^+/−^) mice, which express the A53T mutant of human α-synuclein and firefly luciferase. After a single intraperitoneal injection with α-synuclein fibrils, four of five mice developed paralysis and α-synuclein pathology in the central nervous system, with a median incubation time of 229 ± 17 days. Diseased mice accumulated aggregates of Sarkosyl-insoluble and phosphorylated α-synuclein in the brain and spinal cord, which colocalized with ubiquitin and p62 and were accompanied by gliosis. In contrast, only one of five mice developed α-synuclein pathology in the central nervous system after intraglossal injection with α-synuclein fibrils, after 285 days. These findings are novel and important because they show that, similar to prions, α-synuclein prionoids can neuroinvade the central nervous system after intraperitoneal or intraglossal injection and can cause neuropathology and disease.

**IMPORTANCE** Synucleinopathies are neurodegenerative diseases that are characterized by the pathological presence of aggregated α-synuclein in cells of the nervous system. Previous studies have shown that α-synuclein aggregates made of recombinant protein or derived from brains of patients can spread in the central nervous system in a spatiotemporal manner when inoculated into the brains of animals and can induce pathology and neurologic disease, suggesting that misfolded α-synuclein can behave similarly to prions. Here we show that α-synuclein inoculation into the peritoneal cavity or the tongue in mice overexpressing α-synuclein causes neurodegeneration after neuroinvasion from the periphery, which further corroborates the prionoid character of misfolded α-synuclein.

## INTRODUCTION

α-Synuclein is a soluble, cellular protein that has been identified in an atypical, misfolded state in cellular deposits for a number of neurodegenerative diseases, which have collectively been termed synucleinopathies because of this commonality (1–3). These diseases include, among others, Parkinson's disease (PD), dementia with Lewy bodies (DLB), and multiple system atrophy (MSA). A growing body of evidence shows that α-synuclein has many characteristics that are similar to those of the prion protein. In humans, missense mutations in the *SNCA* gene, encoding α-synuclein, and mutations resulting in elevated protein levels of wild-type α-synuclein cause PD (4–7). α-Synuclein pathology spreads progressively along interconnected areas within the brains of PD patients and has been classified into stages (8, 9). Deposits of misfolded α-synuclein have been discovered in therapeutically grafted embryonic dopaminergic neurons in the striata of PD patients, suggesting that misfolded α-synuclein may spread between cells and seed aggregation of native α-synuclein (10, 11). Besides these observations in human patients, additional evidence from experiments performed *in vitro*, in cell culture, and in animal models also illustrates that misfolded α-synuclein behaves similarly to prions in its capacity to seed misfolding of native α-synuclein, to propagate α-synuclein misfolding between cells, to spread within the nervous system, and to cause neuropathology and disease (12–20). Despite the many parallels between α-synuclein and the prion protein, however, prions are foremost known to be infectious proteins which are naturally transmissible between hosts, causing diseases such as scrapie (among sheep) and chronic wasting disease (CWD) (among deer and elk) (21, 22). Because misfolded α-synuclein does not seem to be transmitted naturally between humans, it has been suggested to refer to the “prion-like” property of misfolded α-synuclein as “prionoid” to emphasize that synucleinopathies are not naturally transmissible between humans (23).

Iatrogenic transmission of Creutzfeldt-Jakob disease (CJD) after treatment of stunted growth in children with cadaveric growth hormone, transmission of bovine spongiform encephalopathy (BSE) from cattle to humans in the form of variant CJD (vCJD) through consumption of BSE-tainted meat products, and transmission of vCJD among humans via blood transfusions have clearly demonstrated that prions can invade the central nervous system (CNS) after peripheral challenge in humans (24–26). Recently, one study also suggested iatrogenic transmission of β-amyloid pathology and cerebral amyloid angiopathy together with CJD after treatment of individuals with human cadaveric growth hormone contaminated with prions (27). We were interested in whether misfolded α-synuclein may have similar prionoid properties and may invade the CNS after peripheral challenge. To address this question, we injected recombinant fibrils of human wild-type α-synuclein or phosphate-buffered saline (PBS) (as a control) into the peritoneal cavity or tongue of bigenic Tg(M83^+/−^:*Gfap*-luc^+/−^) mice, which express the A53T mutant of human α-synuclein and firefly luciferase. Whereas none of the PBS-injected control mice developed signs of disease or neuropathology, we could detect neuroinvasion of α-synuclein prionoids in four of five mice after intraperitoneal challenge and in one of five mice after intraglossal challenge with α-synuclein fibrils. Our results show that injection of α-synuclein fibrils into the peritoneal cavity or tongue can be sufficient for α-synuclein prionoids to invade the CNS and to cause neurodegeneration in Tg(M83^+/−^:*Gfap*-luc^+/−^) mice, corroborating the hypothesis that α-synuclein prionoids share more characteristics with prions than previously anticipated.

## MATERIALS AND METHODS

### Mouse husbandry and inoculations.

B6;C3-Tg(*Prnp*-SNCA*A53T)83Vle/J mice (TgM83^+/−^ mice; The Jackson Laboratory) were crossed to Tg(*Gfap*-luc^+/−^) mice and the progeny genotyped by real-time and standard PCR for the presence of transgenes encoding human α-synuclein with the familial A53T mutation and firefly luciferase (28, 29). Six- to 8-week-old hemizygous, bigenic Tg(M83^+/−^:*Gfap*-luc^+/−^) mice or monogenic Tg(*Gfap*-luc^+/−^) mice were anesthetized with isoflurane and injected using a 27-gauge disposable hypodermic syringe. For intraglossal challenge, Tg(M83^+/−^:*Gfap*-luc^+/−^) mice and Tg(*Gfap*-luc^+/−^) mice were injected with 5 μl of sonicated α-synuclein fibrils or PBS. For intraperitoneal challenge, Tg(M83^+/−^:*Gfap*-luc^+/−^) mice were injected with 50 μl of sonicated α-synuclein fibrils or PBS. After inoculation, animals were monitored daily for health and three times weekly for signs of neurologic disease, such as reduced grooming, ataxia, tremor, bradykinesia, akinesia, lethargy, circling, tail rigidity, paraparesis, paralysis, kyphosis, and more. Mice were narcotized with isoflurane and then transcardially perfused with 0.9% (wt/vol) saline followed by 10% formalin neutral buffer solution (Sigma). Brains were fixed overnight with 10% formalin neutral buffer solution for immunohistochemistry. For biochemical analysis, brains were snap-frozen on dry ice and stored at −80°C. The mice were housed under standard conditions with a 12-h light-dark cycle and free access to food and water. All animal studies were approved by the animal protection committee of the North Rhine-Westphalia State Environment Agency (LANUV).

### Preparation of recombinant α-synuclein protein fibrils.

Fibrils of human and mouse wild-type α-synuclein were prepared as previously described (20). Mouse wild-type α-synuclein was purchased (Mybiosource), and human wild-type α-synuclein was prepared from Escherichia coli. Briefly, E. coli cells harboring the pET-3a expression plasmid (Novagen) for human wild-type α-synuclein were grown at 37°C in 1 liter of LB medium containing ampicillin, chloramphenicol, and 1% glucose to an optical density at 600 nm (OD_600_) of 0.5, induced with 0.1 mM isopropyl-β-d-thiogalactopyranoside (IPTG), and grown for 5 h at 37°C. Periplasmic material was released into the buffer by osmotic shock, and the cells were pelleted by centrifugation at 6,000 × *g* for 15 min. The pellet was resuspended in 35% sucrose solution in 2 mM EDTA and 30 mM Tris-HCl (pH 7.2) and incubated with shaking at room temperature for 15 min. The cells were again harvested and resuspended in ice-cold water containing 5 mM MgSO_4_. The periplasmic material was boiled for 20 min and then centrifuged at 5,000 × *g* for 30 min. The supernatant was subjected to fractional ammonium sulfate precipitation. Briefly, (NH_4_)_2_SO_4_ crystals were added to the supernatant with gentle stirring on ice over 10 min, to 35% saturation (19.4 g/100 ml), after which centrifugation was repeated. (NH_4_)_2_SO_4_ crystals (11.8 g/100 ml) were then added over 10 min to take the concentration from 35% to 55% saturation, with gentle stirring on ice, after which centrifugation was repeated. The pellet was resuspended in 10 ml water and dialyzed three times for 3 h against 20 mM Tris-HCl (pH 8.0). α-Synuclein was purified from the supernatant by Resource Q anion-exchange chromatography, using 20 mM Tris-HCl (pH 8.0) as binding buffer and 500 mM NaCl in 10 mM Tris-HCl (pH 8.0) as elution buffer on an Äkta Pure chromatography system (GE Healthcare). α-Synuclein was released from the column by use of a 30-ml linearly increasing gradient from the binding buffer toward the elution buffer and then dialyzed against 150 mM NaCl in 20 mM Tris-HCl (pH 7.2). Fibril assembly for α-synuclein was performed in an orbital thermomixer (Eppendorf) agitating 3 μg/μl protein at 800 rpm and 37°C for 5 days. Fibrils were diluted in PBS and sonicated for 1 min with pulses of 1 s by use of a Sonoplus Mini20 sonicator (Bandelin) prior to injection.

### Negative-stain electron microscopy.

Transmission electron microscopy (TEM) was performed using a Tecnai F20 TEM (FEI Company) operating at an acceleration voltage of 200 kV. Five-microliter samples were adsorbed for 30 s onto freshly glow-discharged Formvar-carbon-coated 200-mesh copper grids. The grids were washed briefly with 0.1 M and 0.01 M ammonium acetate buffer (pH 7.4) and then stained with two 50-μl drops of freshly filtered 2% (wt/vol) uranyl acetate. The grids were allowed to dry overnight before viewing, and the electron micrographs were recorded on an Eagle 4K charge-coupled device (CCD) camera (FEI Company). Three different preparations of α-synuclein were characterized by thoroughly inspecting at least five different areas per grid.

### Bioluminescence imaging.

For noninvasive visualization of the bioluminescence signals from the brains of injected mice, animals were imaged every 2 to 4 weeks with an IVIS Lumina II imaging system (PerkinElmer). Prior to imaging the scalp, hair was shaved and depilated with a depilatory cream. To block unspecific bioluminescence from the ears, the ears were colored black. The substrate for luciferase, d-luciferin potassium salt (Acris), was diluted in PBS and injected intraperitoneally at 150 mg per kg of body weight. Mice were anesthetized with an isoflurane-oxygen gas mix applied by use of an evaporator (2 liters/min), and after 10 min of incubation, they were imaged for 60 s. Bioluminescence images were quantitated with Living Image *in vivo* imaging software 3.0 (PerkinElmer).

### Immunohistochemical analysis.

Brains of PBS- and formalin neutral buffer solution-perfused mice were fixed in formalin overnight, dehydrated in a series of alcohol baths, and embedded in paraffin. Brains were cut into 8-μm-thick coronal sections, mounted on glass slides, deparaffinized in two xylol baths for 5 to 10 min, and finally rehydrated through a series of graded ethanol baths. For antigen retrieval, slides were incubated in citrate buffer (pH 6.0) for 5 min at room temperature and then boiled for 10 min in a microwave oven. After cooling, slides were incubated with a 3% hydrogen peroxide solution for 30 min to inhibit endogenous peroxidases. Slides were blocked with a buffer containing 20% (vol/vol) normal goat serum, 1% (vol/vol) bovine serum albumin (BSA), and 0.5% Triton X-100 in PBS for 1 h at room temperature and then incubated with a primary antibody diluted in 1% (vol/vol) normal goat serum, 1% (vol/vol) BSA, and 0.25% Triton X-100 in PBS overnight at 4°C. The antibodies used in this study and their corresponding dilutions are listed in Table 1. After washing twice with 0.25% (vol/vol) Triton X-100 in PBS and once with PBS, sections were incubated with a peroxidase-conjugated secondary antibody by use of a Vectastain ABC or MOM kit (Vector Laboratories). Peroxidase-positive structures were visualized by incubation with DAB (3,3′-diaminobenzidine) for 15 to 30 s. To inactivate the oxidation process, the slides were shortly transferred to a 3% hydrogen peroxide bath. After counterstaining of acidic and negatively charged structures with hematoxylin QS (Vector Laboratories), the slides were coverslipped with Vectamount AQ (Vector Laboratories). Visual analysis was performed with Zen Lite software (Carl Zeiss) after scanning of the slides with an Axio Scan.Z1 slide scanner (Carl Zeiss).

### Immunofluorescence analysis.

Paraffin-embedded tissues were cut into 8-μm-thick coronal sections, mounted on glass slides, deparaffinized, and rehydrated as described above. For antigen retrieval, the slides were incubated in citrate buffer (pH 6.0) for 5 min at room temperature and additionally boiled for 10 min in a microwave oven. After cooling, the slides were blocked in 20% (vol/vol) normal goat serum, 1% (vol/vol) BSA, and 0.5% (vol/vol) Triton X-100 in PBS for 1 h at room temperature. Sections were incubated with a primary antibody in 1% (vol/vol) normal goat serum, 1% (vol/vol) BSA, and 0.25% Triton X-100 in PBS overnight at 4°C. Antibodies used in this study and their corresponding dilutions are listed in Table 1. After washing twice with 0.25% (vol/vol) Triton X-100 in PBS and once with PBS, sections were stained with corresponding Alexa Fluor 488- or Alexa Fluor 594-conjugated (Thermo Fisher Scientific) secondary antibodies and the nuclear dye DAPI (4′,6-diamidino-2-phenylindole; Thermo Fisher Scientific) in 1% (vol/vol) normal goat serum, 1% (vol/vol) BSA, and PBS for 1 h at room temperature. Slides were coverslipped with Fluoromount medium (Sigma) and visualized with an LSM700 confocal laser scanning microscope (Carl Zeiss).

**TABLE 1:** Antibodies used for immunofluorescence, immunohistochemistry, and Western blot assays

Target (abbreviation) [antibody clone]	Source	Host	Immunogen	Dilution^*a*^	
				IHC/IF	WB
Actin [C4]	Abcam	Mouse			1:1,000
α-Synuclein, phospho-S129 [81A]	Biolegend	Mouse	pSer129	1:200	
α-Synuclein, phospho-S129 [pSyn#64]	Wako	Mouse	pSer129	1:1,200	
α-Synuclein, phospho-S129 [EP1536Y]	Abcam	Rabbit	pSer129	1:100	1:1,000
Human α-synuclein [Syn211]	Merck Millipore	Mouse	121–125	1:100	
Mouse α-synuclein [D37A6]	Cell Signaling	Rabbit	103–110	1:100	
Choline *O*-acetyltransferase (ChAT)	Merck Millipore	Rabbit		1:100	
Glial fibrillary acidic protein (GFAP)	Dako	Rabbit		1:200	
IBA-1	Wako	Rabbit		1:500	
Sequestosome-1 (p62)	Proteintech	Rabbit		1:100	
Ubiquitin [Ubi-1]	Merck Millipore	Mouse		1:500	

a IHC, immunohistochemistry; IF, immunofluorescence; WB, Western blotting.

### Biochemical analysis.

Brain or spinal cord samples were homogenized in Ca^2+^- and Mg^2+^-free PBS (pH 7.4) in the presence of protease and phosphatase inhibitors (HALT protease and phosphatase inhibitor cocktail; Thermo Fisher Scientific) by two 30-s cycles in a Precellys 24-Dual homogenizer (Peqlab) to reach a final concentration of 20% (wt/vol) for brain homogenates and 10% (wt/vol) for spinal cord homogenates, which were afterwards sonicated twice for 10 s. After adjusting homogenates to 750 mM NaCl, the samples were centrifuged at 1,000 × *g* for 5 min at 4°C. For further analysis, 1.0 mg of total protein from brain homogenates or 0.8 mg from spinal cord homogenates was incubated on ice for 15 min in *N*-lauroylsarcosyl (Sigma) at a final concentration of 10% (wt/vol). Homogenates were ultracentrifuged at 465,000 × *g* for 1 h at 4°C over a 3-ml 10% (wt/vol) sucrose cushion in a TLA-110 rotor (Beckman Coulter). Pellets were resuspended in 45 μl of TD4215 denaturing buffer containing 4% sodium dodecyl sulfate (SDS), 2% β-mercaptoethanol, 192 mM glycine, 25 mM Tris, and 5% (wt/vol) sucrose. For heat denaturation, samples were boiled at 100°C for 5 min and loaded onto 4 to 12% NuPage gels (Thermo Fisher Scientific). SDS-polyacrylamide gels were processed in a morpholineethanesulfonic acid (MES) buffer system (Thermo Fisher Scientific). Separated proteins were transferred to polyvinylidene difluoride (PVDF) membranes by use of a semidry blotting system and then cross-linked with a 0.4% (vol/vol) formalin solution in PBS for 30 min on a rotor at room temperature. Membranes were blocked in a buffer containing Tris-buffered saline (TBS), 0.05% (vol/vol) Tween 20, and 5% (wt/vol) milk for 1 h at room temperature. Blots were subsequently probed with primary antibody overnight at 4°C. The primary antibody for phosphorylated α-synuclein was detected with a horseradish peroxidase-conjugated secondary antibody (Cayman) by 1 h of incubation at room temperature. The chemiluminescence reaction was visualized with SuperSignal West Dura extended-duration substrate (Thermo Fisher Scientific) in a chemiluminescence reader (Stella; Raytek). The primary antibody for actin was detected with an IRDye 680-conjugated antibody (Li-Cor Biosciences) and visualized with an Odyssey infrared imaging system (Li-Cor Biosciences).

## RESULTS

### Bigenic Tg(M83^+/−^:*Gfap*-luc^+/−^) mice develop neurologic disease after intraperitoneal and intraglossal challenge with α-synuclein fibrils.

**FIG 1:**
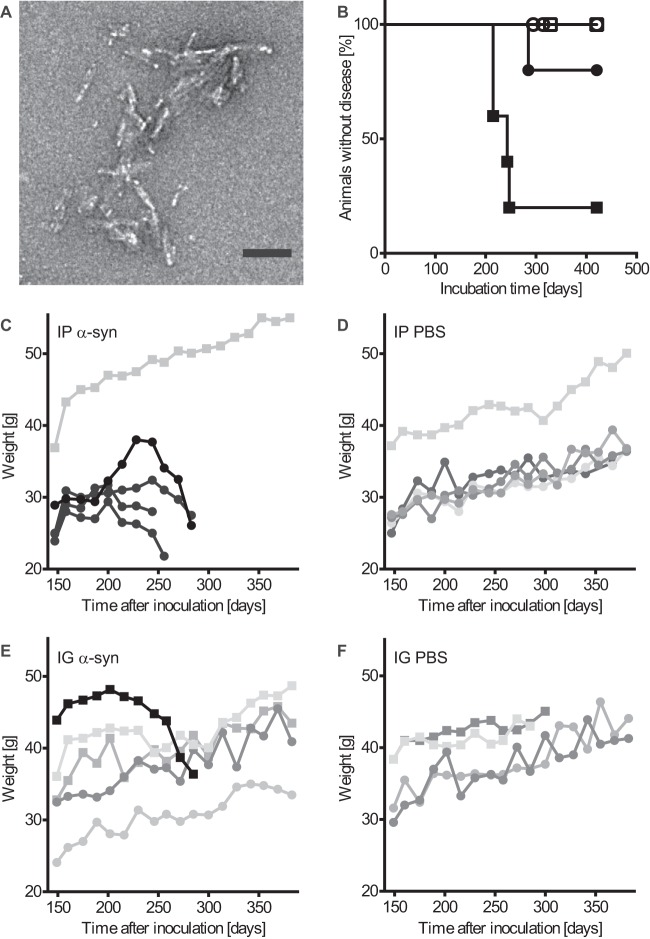
Challenge with α-synuclein fibrils causes disease in bigenic Tg(M83^+/−^:*Gfap*-luc^+/−^) mice. (A) Negatively stained electron micrograph of sonicated recombinant human α-synuclein fibrils that were injected into mice. Aggregated samples were stained with uranyl acetate. Multiple clusters of short, rod-shaped aggregates are visible. Bar = 100 nm. (B) Kaplan-Meier survival curves show that after intraperitoneal injection with α-synuclein fibrils, four of five Tg(M83^+/−^:*Gfap*-luc^+/−^) mice developed signs of neurologic dysfunction in 229 ± 17 days (black squares), whereas none of the PBS-injected control mice developed neurologic disease within 420 days (white squares). One mouse of five died 285 days after intraglossal inoculation with α-synuclein fibrils (black circles). In contrast, none of the PBS-injected mice developed neurologic disease or spontaneously died (white circles). (C) Four (black symbols) of five mice that had been inoculated intraperitoneally (IP) with α-synuclein (α-syn) fibrils continuously lost weight for 6 to 8 weeks before they developed signs of neurologic disease. The one surviving animal continuously gained weight and did not develop disease or pathology for up to 420 days after inoculation (gray symbols). (E) One (black symbols) of five mice inoculated intraglossally (IG) with α-synuclein fibrils continuously lost weight for at least 8 weeks before it died. The four mice that did not succumb to disease or die continued to gain weight (gray symbols). Mice injected intraperitoneally (D) or intraglossally (F) with PBS gained weight throughout the course of the experiment. In panels C to F, male mice are represented by squares and female mice by circles.

We crossed TgM83^+/−^ mice, expressing the A53T mutant of human α-synuclein from the *Prnp* promoter, with Tg(*Gfap*-luc^+/−^) reporter mice, expressing firefly luciferase from the promoter for glial fibrillary acidic protein (GFAP), to obtain bigenic Tg(M83^+/−^:*Gfap*-luc^+/−^) mice that express both transgenes hemizygously (28, 29). Tg(M83^+/−^:*Gfap*-luc^+/−^) mice and TgM83^+/−^ mice remained free of spontaneous neurologic disease and pathology for over 650 days, as also reported by others (17). Inoculation of prions into the peritoneal cavity or, especially, into the tongue efficiently transmits prion disease to rodents (30). To test bigenic Tg(M83^+/−^:*Gfap*-luc^+/−^) mice for susceptibility to peripheral challenge via these routes, we injected 6- to 8-week-old mice with recombinant human wild-type α-synuclein fibrils via the peritoneal cavity (50 μg) or the tongue (10 μg) (Fig. 1A and Table 2). Control mice were injected with PBS. Whereas none of the PBS-inoculated mice developed disease throughout the course of the experiment (420 days), four of five mice inoculated intraperitoneally with α-synuclein fibrils developed neurologic disease, with marked signs of paralysis, kyphosis, and reduced activity, within 229 ± 17 days (mean ± standard deviation [SD]) after inoculation (Fig. 1B; see Movie S1 in the supplemental material). In addition, the four diseased mice also started to lose weight beginning 6 to 8 weeks before they became terminally sick (Fig. 1C). Only one Tg(M83^+/−^:*Gfap*-luc^+/−^) mouse out of five died, at 285 days, after intraglossal inoculation with α-synuclein fibrils, after continuously losing weight for 8 weeks (Fig. 1B and E). In contrast, none of the PBS-injected mice lost weight for prolonged periods before being sacrificed 420 days after inoculation (Fig. 1D and F). Because an even smaller amount of wild-type mouse α-synuclein fibrils (5 μg) was reported to induce neurodegeneration in wild-type mice after intracerebral inoculation (15), we wanted to investigate if the same amount of mouse α-synuclein fibrils would also cause neurodegeneration after intraglossal inoculation of mice not overexpressing α-synuclein. We intraglossally injected monogenic Tg(*Gfap*-luc^+/−^) mice, which express only the endogenous mouse α-synuclein, with 5 μg of wild-type mouse α-synuclein fibrils or PBS (Table 2). In contrast to intracerebral inoculation of wild-type mice (15), intraglossal inoculation of Tg(*Gfap*-luc^+/−^) mice did not cause neurodegeneration.

**TABLE 2:** Inoculation experiments

Mouse line	Inoculum (amt [μg])	Inoculation route	No. of mice with disease/no. of mice inoculated	Mean ± SD survival time (days)
Tg(M83^+/−^:*Gfap*-luc^+/−^) mice	Human α-synuclein fibrils (50)	Intraperitoneal	4/5	229 ± 17
	PBS	Intraperitoneal	0/5	420
	Human α-synuclein fibrils (10)	Intraglossal	1/5	285
	PBS	Intraglossal	0/4	420
Tg(*Gfap*-luc^+/−^) mice	Mouse α-synuclein fibrils (5)	Intraglossal	0/8	420
	PBS	Intraglossal	0/9	420

### Diseased Tg(M83^+/−^:*Gfap*-luc^+/−^) mice accumulate aggregated species of phosphorylated α-synuclein in the CNS.

**FIG 2:**
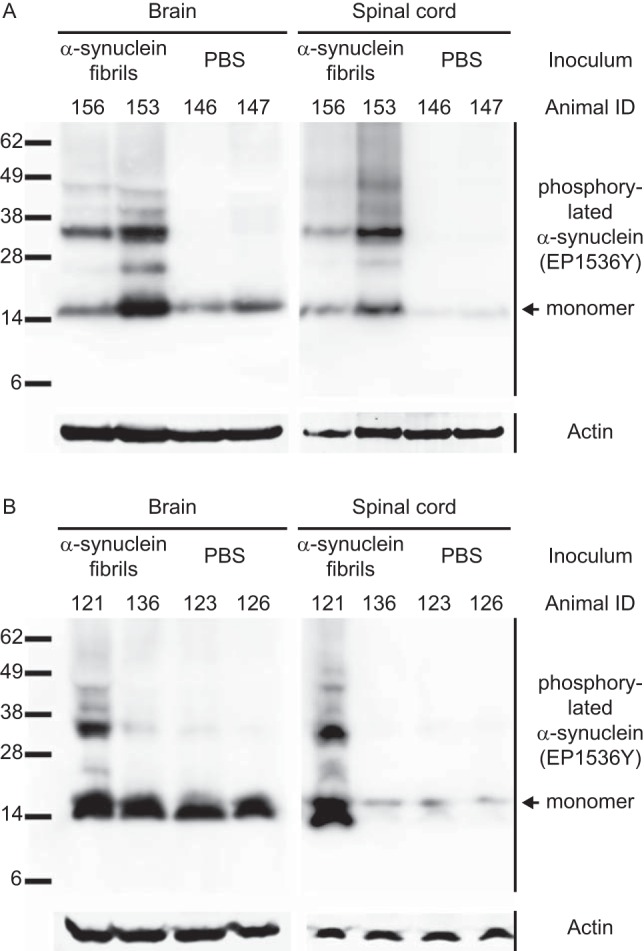
Biochemical analysis of phosphorylated α-synuclein in the CNS of peripherally injected Tg(M83^+/−^:*Gfap*-luc^+/−^) mice. (A) Detection with the EP1536Y antibody, which recognizes phosphorylation at Ser129 of α-synuclein, showed that diseased mice accumulated high-molecular-weight species of Sarkosyl-insoluble aggregates of phosphorylated α-synuclein in their brains and spinal cords, whereas control mice challenged with PBS did not and showed bands only for the monomeric form of phosphorylated α-synuclein. (B) After intraglossal challenge with α-synuclein fibrils, only one mouse, animal 121, accumulated Sarkosyl-insoluble aggregates of phosphorylated α-synuclein in the brain and spinal cord, whereas all other intraglossally inoculated animals remained healthy. Molecular masses are shown in kilodaltons. Sample loading in each lane is shown by detection of actin.

Pathological deposits of misfolded α-synuclein are often accompanied by posttranslational modifications of α-synuclein, such as increased phosphorylation, in particular at Ser129, which has frequently been used to characterize the extent of neuropathology in the brains of PD patients and in animal models of synucleinopathies (9, 16, 31). Biochemical analysis of tissue homogenates from Tg(M83^+/−^:*Gfap*-luc^+/−^) mice intraperitoneally injected with α-synuclein fibrils showed that diseased mice had accumulated Sarkosyl-insoluble aggregates of phosphorylated α-synuclein in their brains and spinal cords as determined by probing with the EP1536Y antibody, which recognizes phosphorylation at Ser129 of α-synuclein (Fig. 2A). These aggregates presented as several additional bands above the 15-kDa band of monomeric, phosphorylated α-synuclein. In contrast, the brains and spinal cords of PBS-injected Tg(M83^+/−^:*Gfap*-luc^+/−^) mice remained free of Sarkosyl-insoluble aggregates and showed staining only for the monomeric form of phosphorylated α-synuclein (Fig. 2A). Also, the mouse that died 285 days after intraglossal injection with α-synuclein fibrils showed higher-molecular-weight bands characteristic of phosphorylated α-synuclein pathology, but the mice that did not develop disease and the control mice that were injected with PBS did not (Fig. 2B). Equally important, none of the intraglossally inoculated monogenic Tg(*Gfap*-luc^+/−^) mice had accumulated aggregated species of phosphorylated α-synuclein in the CNS 420 days after inoculation.

### Deposits of phosphorylated α-synuclein are widely distributed in the brains and spinal cords of diseased Tg(M83^+/−^:*Gfap*-luc^+/−^) mice.

**FIG 3:**
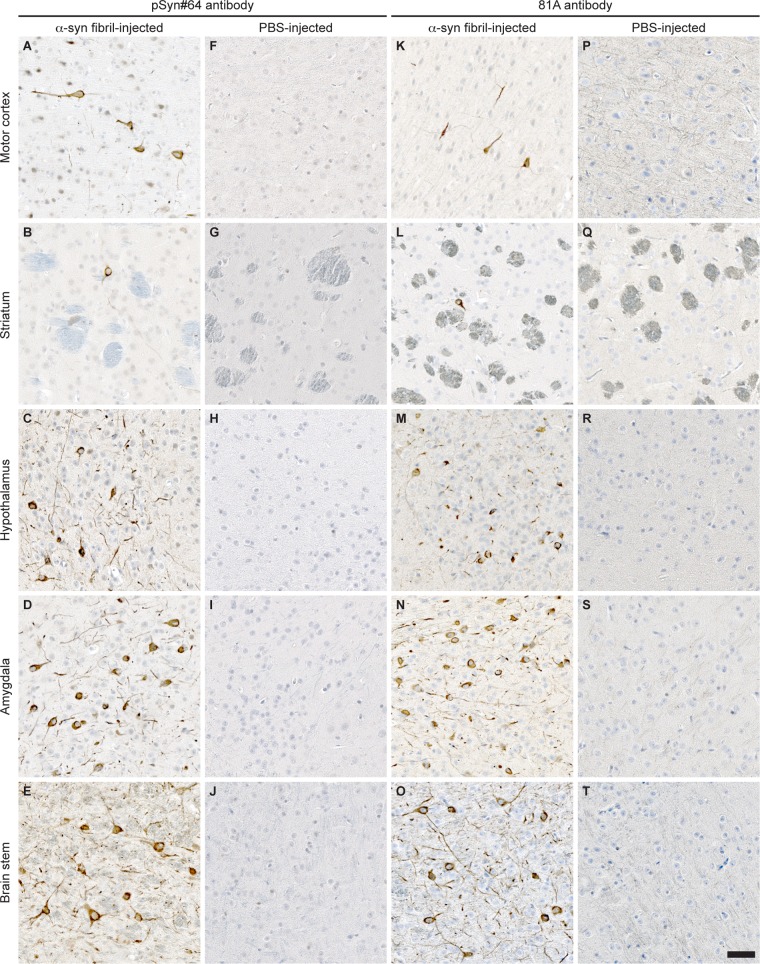
Immunohistochemical analysis shows neuropathology in brains of Tg(M83^+/−^:*Gfap*-luc^+/−^) mice after intraperitoneal injection with α-synuclein fibrils. (A to E and K to O) Brain sections from animals injected with α-synuclein fibrils accumulated deposits of phosphorylated α-synuclein in multiple brain regions, as detected with the pSyn#64 antibody (A to E) and the 81A antibody (K to O), which recognize phosphorylation at Ser129 of α-synuclein. (F to J and P to T) In contrast, none of the PBS-injected mice showed any detectable aggregates of phosphorylated α-synuclein in the brain. Bar = 50 μm.

**FIG 4:**
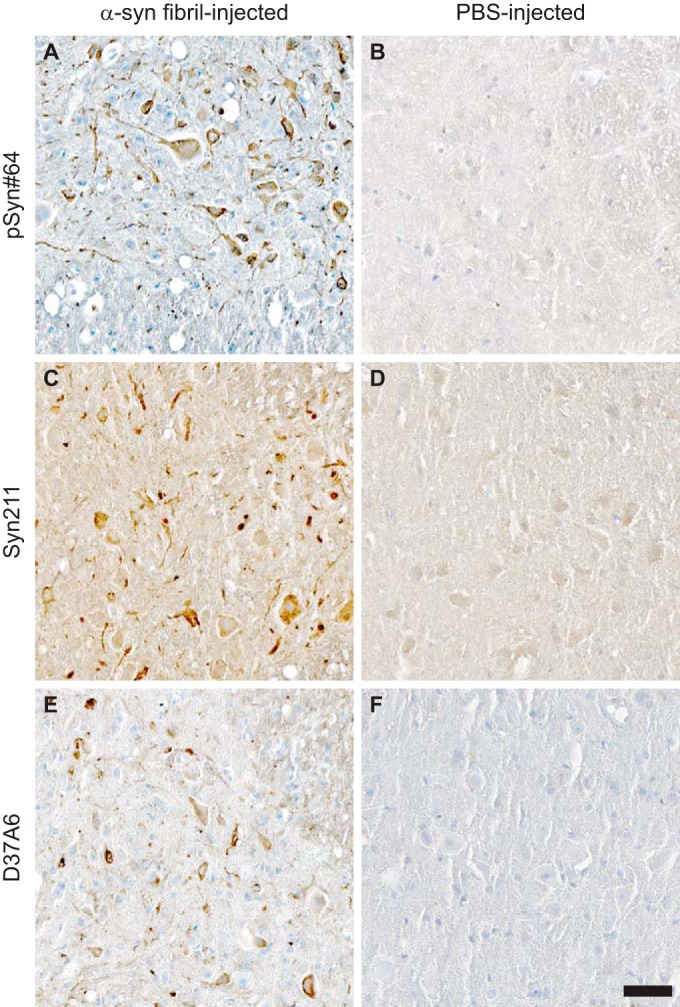
Immunohistochemistry shows abnormal, phosphorylated α-synuclein deposits in spinal cords of Tg(M83^+/−^:*Gfap*-luc^+/−^) mice after intraperitoneal injection with α-synuclein fibrils. (A) Deposits of phosphorylated α-synuclein were present in spinal cord sections from diseased animals, as illustrated by staining with the pSyn#64 antibody. (C and E) These deposits were composed of transgenically expressed human A53T α-synuclein, as shown with the Syn211 antibody (C), and endogenously expressed mouse α-synuclein, as visualized with the D37A6 antibody (E). (B, D, and F) None of the PBS-injected animals accumulated deposits of aggregated α-synuclein in the spinal cord. Bar = 50 μm.

**FIG 5:**
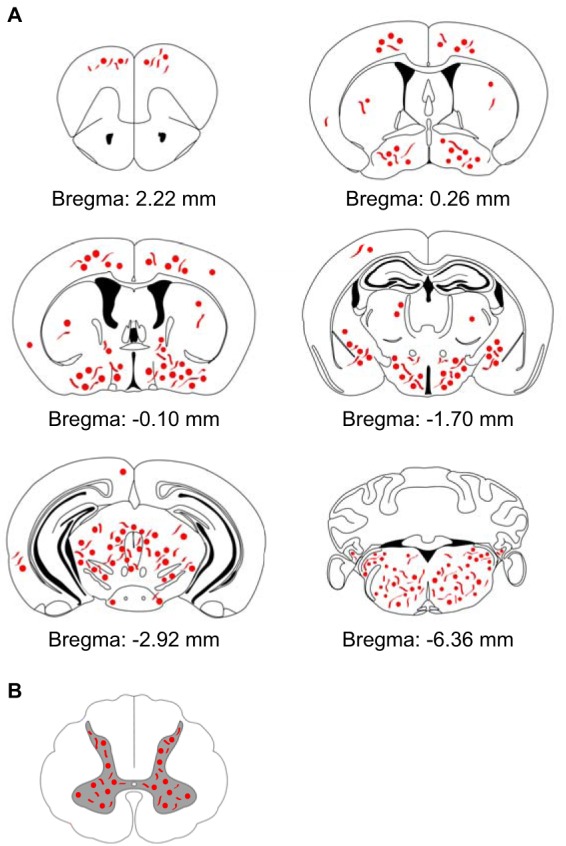
Schematic overview showing the distribution of deposits of phosphorylated α-synuclein in the CNS of diseased Tg(M83^+/−^:*Gfap*-luc^+/−^) mice after intraperitoneal challenge with α-synuclein fibrils. (A) The distributions of abnormal deposits detected by use of two different antibodies, pSyn#64 and 81A, were comparable, and deposits could be found almost throughout the brain. However, the cerebellum was devoid of abnormal deposits of phosphorylated α-synuclein. (B) Abnormal aggregates of phosphorylated α-synuclein were also present in neurons within the gray matter of the spinal cords of diseased animals.

**FIG 6:**
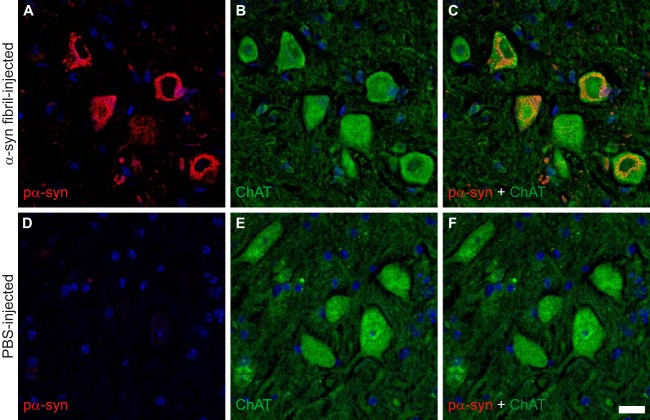
Phosphorylated α-synuclein can be detected in motor neurons of diseased Tg(M83^+/−^:*Gfap*-luc^+/−^) mice. (A to C) Immunofluorescence analysis showed that deposits of phosphorylated α-synuclein, as stained with the pSyn#64 antibody, were widespread in the gray matter and could also be detected in motor neurons within the ventral horn of the spinal cord in diseased Tg(M83^+/−^:*Gfap*-luc^+/−^) mice. (D to F) PBS-injected healthy control animals did not show aggregates of phosphorylated α-synuclein in their motor neurons. Motor neurons were detected with an antibody to choline *O*-acetyltransferase (ChAT). Nuclear staining with DAPI is shown in blue. Bar = 20 μm.

Immunohistochemical staining with two different antibodies against phosphorylated α-synuclein, pSyn#64 (Fig. 3A to J) and 81A (Fig. 3K to T), revealed abundant deposits in neuronal cell bodies and neurites in the brains (Fig. 3A to E and K to O) and spinal cords (Fig. 4A) of diseased animals but not the brains (Fig. 3F to J and P to T) or spinal cords (Fig. 4B) of PBS-injected control animals. Equally important, fibril-injected animals surviving throughout the course of the experiment, until 420 days after inoculation, remained free of deposits of phosphorylated α-synuclein. In diseased animals, deposits of phosphorylated α-synuclein were widespread throughout the cerebrum but absent in the cerebellum (Fig. 5). In the spinal cord, neuronal deposits of phosphorylated α-synuclein were widely distributed in the gray matter and could also be detected in motor neurons in the ventral horns of diseased animals (Fig. 6A to C) but not in motor neurons of PBS-injected control animals (Fig. 6D to F). Deposits of aggregated α-synuclein in the CNS of diseased animals not only consisted of transgenically expressed mutant human α-synuclein as detected with the Syn211 antibody (Fig. 4C), which is specific for human α-synuclein, but also consisted of aggregates of endogenously expressed mouse α-synuclein as detected with the D37A6 antibody, which is specific for mouse α-synuclein (Fig. 4E). Both antibodies, Syn211 and D37A6, did not reveal any α-synuclein deposits in the CNS of PBS-injected control mice (Fig. 4D and F).

### Deposits of phosphorylated α-synuclein colocalize with ubiquitin and p62.

**FIG 7:**
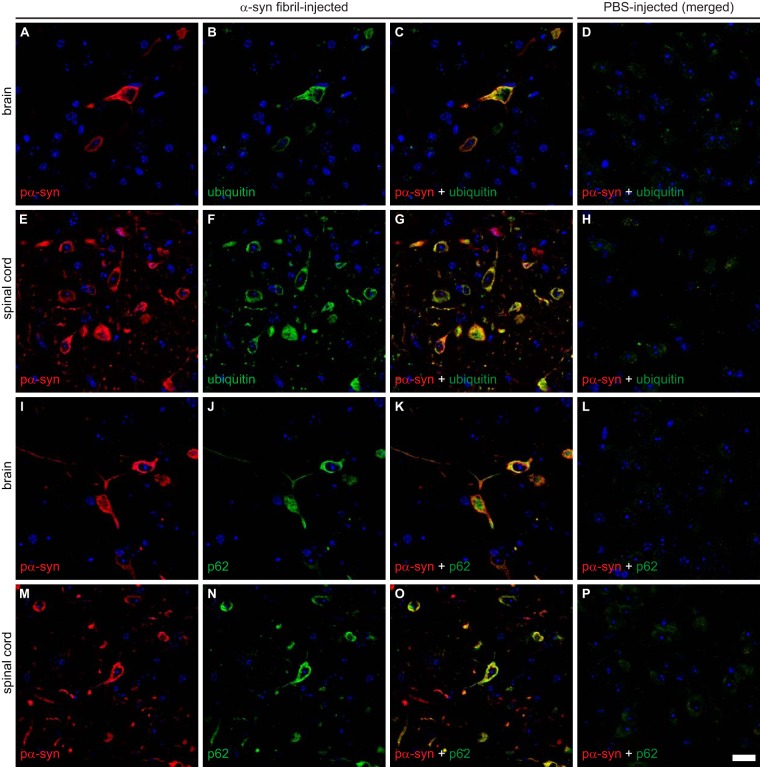
Immunofluorescence analysis shows that deposits of phosphorylated α-synuclein colocalize with ubiquitin and p62 in the brains and spinal cords of diseased Tg(M83^+/−^:*Gfap*-luc^+/−^) mice. Phosphorylated α-synuclein, detected with the EP1536Y antibody, and ubiquitin colocalized in the brains (A to C) and spinal cords (E to G) of fibril-injected diseased mice. Similarly, phosphorylated α-synuclein, detected here with the pSyn#64 antibody, also colocalized with p62 in brains (I to K) and spinal cords (M to O) of diseased mice. (D, H, L, and P) PBS-injected healthy control animals did not show aggregation or colocalization for any of these proteins. Nuclear staining with DAPI is shown in blue. Bar = 20 μm.

Ubiquitin and p62 (also known as sequestosome-1) are involved in protein degradation and often associate with pathological protein deposits that seem to be resistant to degradation, such as α-synuclein in Lewy bodies of patients with PD, in glial cytoplasmic inclusions in patients with MSA, and in animal models of synucleinopathies (18, 32, 33). To further characterize the nature of α-synuclein-positive deposits in the CNS of diseased Tg(M83^+/−^:*Gfap*-luc^+/−^) mice, we performed immunofluorescence staining of brain and spinal cord sections for phosphorylated α-synuclein and ubiquitin or phosphorylated α-synuclein and p62 (Fig. 7). We found deposits of phosphorylated α-synuclein to colocalize with ubiquitin and p62 in the brains and spinal cords of diseased Tg(M83^+/−^:*Gfap*-luc^+/−^) mice, which is indicative of aberrant protein homeostasis for α-synuclein. PBS-injected healthy control animals did not show aggregates or colocalization for any of these proteins (Fig. 7D, H, L, and P).

### Diseased mice develop reactive astrogliosis and microgliosis.

**FIG 8:**
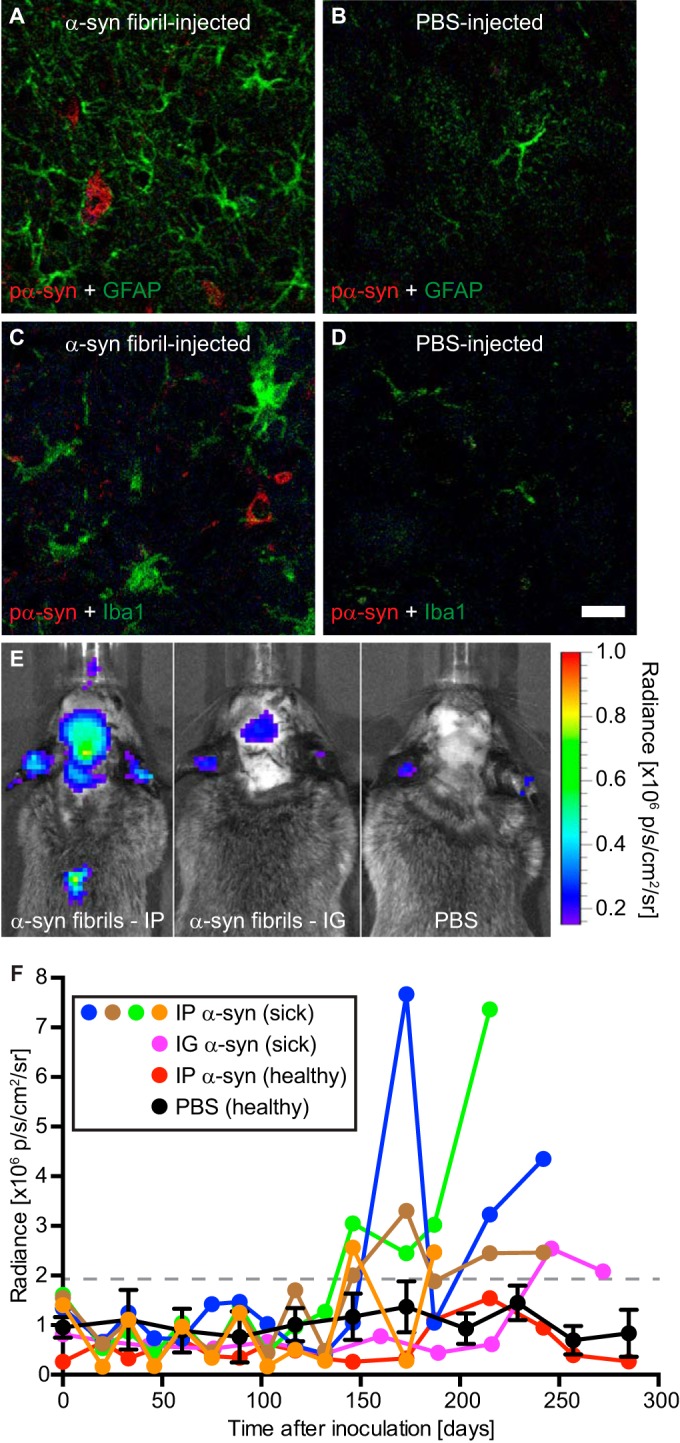
Gliosis in the brains of diseased Tg(M83^+/−^:*Gfap*-luc^+/−^) mice. (A) Immunofluorescence analysis of brain sections with an antibody against GFAP, a marker of astrocytes, showed that diseased animals had reactive astrogliosis in areas with deposits of phosphorylated α-synuclein, which were detected in the brain stem by use of the pSyn#64 antibody. (B) In contrast, reactive astrogliosis was not observed in brains of healthy, PBS-injected control mice. (C and D) Similarly, staining with an antibody to IBA-1, a marker of microglia, showed that diseased animals had microgliosis in areas with deposits of phosphorylated α-synuclein (C), which was not observed in brains of healthy, PBS-injected control mice (D). (E) Bioluminescence imaging revealed that Tg(M83^+/−^:*Gfap*-luc^+/−^) mice that had been challenged intraperitoneally with α-synuclein fibrils showed elevated radiance from their brains and spinal cords (left panel), caused by the increased activation of astrocytes, shortly before they developed neurologic symptoms, but this was not observed in PBS-injected control mice (right panel). After intraglossal inoculation with α-synuclein fibrils, one Tg(M83^+/−^:*Gfap*-luc^+/−^) mouse, animal 121, showed signs of reactive astrogliosis shortly before it died, at 285 days (center panel). p, photons. (F) After intraperitoneal challenge of Tg(M83^+/−^:*Gfap*-luc^+/−^) mice with α-synuclein fibrils, we measured increased levels of bioluminescence (>2 × 10^6^ p/s/cm^2^/sr) from the brains of four mice (blue, green, brown, and orange circles) shortly before they developed neurologic signs of disease. One animal that was intraglossally injected with α-synuclein fibrils also showed elevated levels of bioluminescence before it died 285 days after injection (magenta circles). In contrast, healthy, PBS-injected control mice (black circles; error bars show SD [*n* = 4]) and the animal that did not develop disease after intraperitoneal injection with α-synuclein fibrils (red circles) did not show increased levels of bioluminescence (<2 × 10^6^ p/s/cm^2^/sr) within the period of observation. Bar = 20 μm.

We also investigated whether neurologic disease in the affected Tg(M83^+/−^:*Gfap*-luc^+/−^) mice was accompanied by neuroinflammatory changes. Immunofluorescence staining of brain sections from diseased mice for GFAP, a marker of astrocytes, showed reactive astrogliosis in the presence of deposits of phosphorylated α-synuclein (Fig. 8A). In contrast, healthy, PBS-injected control mice did not show deposits of phosphorylated α-synuclein and equally lacked reactive astrogliosis (Fig. 8B). Since neurodegenerative changes are often accompanied not only by reactive astrogliosis but also by microgliosis, we stained brain sections for IBA-1 (ionized calcium-binding adapter molecule 1), also known as AIF-1 (allograft inflammatory factor 1), a marker of activated microglia. Brain sections from diseased Tg(M83^+/−^:*Gfap*-luc^+/−^) mice again showed abundant activated microglia in the presence of deposits of phosphorylated α-synuclein (Fig. 8C), whereas the brains of PBS-injected control mice were devoid of activated microglia (Fig. 8D). Moreover, bioluminescence imaging of all intraperitoneally inoculated mice revealed increased radiance (>2 × 10^6^ p/s/cm^2^/sr) from the brains of Tg(M83^+/−^:*Gfap*-luc^+/−^) mice injected with α-synuclein fibrils shortly before they developed signs of neurologic disease, but there was no increased radiance from the brains of mice injected with PBS (Fig. 8E and F). Bioluminescence imaging of the mouse that died 285 days after intraglossal injection with α-synuclein fibrils also showed elevated radiance (>2 × 10^6^ p/s/cm^2^/sr) shortly before it died (Fig. 8E and F). Increased radiance in Tg(M83^+/−^:*Gfap*-luc^+/−^) mice is indicative of reactive astrogliosis, since luciferase expression is driven from the *Gfap* promoter. Taken together, the data show that deposition of phosphorylated α-synuclein in Tg(M83^+/−^:*Gfap*-luc^+/−^) mice with neurologic disease is accompanied by reactive gliosis, a hallmark of neuroinflammation.

## DISCUSSION

Our data show that α-synuclein prionoids can invade the CNS after a single injection into the peritoneal cavity or the tongue, resulting in neuropathology in the brain and spinal cord, with widespread deposition of misfolded α-synuclein, and in signs of neuroinflammation and disease in Tg(M83^+/−^:*Gfap*-luc^+/−^) mice. Compared to *bona fide* prions that can invade the CNS after peripheral challenge, similar prionoid behavior was previously reported for misfolded β-amyloid, which induced cerebral amyloidosis after intraperitoneal injection into APP23 mice expressing the human β-amyloid precursor protein with the Swedish mutation (34). Similarly, intraperitoneal injection of aggregated tau seeds into transgenic mice expressing human mutant P301S tau was found to trigger cerebral tauopathy (35). The mean incubation period of ∼229 days that we measured for Tg(M83^+/−^:*Gfap*-luc^+/−^) mice after intraperitoneal challenge with 50 μg of α-synuclein fibrils is surprisingly similar to the incubation time after intracerebral inoculation (∼216 days) of the same mouse line with 30 μl of a 1% brain homogenate from sick TgM83^+/+^ mice, implying that intraperitoneal infection can be very efficient in causing disease (17). Intraglossal inoculation of Tg(M83^+/−^:*Gfap*-luc^+/−^) mice caused neurodegeneration in only one of five mice, and it died at 285 days. This animal had continuously lost weight, had showed signs of astrocytic gliosis, and had accumulated misfolded α-synuclein in its brain and spinal cord before it died, which shows that α-synuclein prionoids had reached and replicated in the CNS after intraglossal challenge. We used only 10 μg of fibrils for intraglossal inoculations because it is more difficult to inoculate large volumes into the tongue than into the peritoneum, and it is possible that a higher inoculum may have led to a higher transmission rate. None of the monogenic Tg(*Gfap*-luc^+/−^) mice developed disease or pathology after intraglossal challenge with α-synuclein fibrils. It is possible that the inoculated amount of α-synuclein fibrils, 5 μg in Tg(*Gfap*-luc^+/−^) mice versus 10 μg in Tg(M83^+/−^:*Gfap*-luc^+/−^) mice, may not have been high enough to induce disease after intraglossal challenge in Tg(*Gfap*-luc^+/−^) mice. However, 5 μg is an amount that readily causes neurodegeneration in wild-type mice after intracerebral inoculation (15). Additional experiments with mice lacking expression of the mutant human A53T-synuclein transgene are warranted to find out whether or not peripheral overexpression of α-synuclein is necessary for α-synuclein prionoids to induce disease after intraperitoneal challenge, which we have not investigated here. After intraglossal inoculation, prions reach the CNS via retrograde axonal transport along the hypoglossal nerve, which is also a likely route for the propagation of α-synuclein prionoids (30); we are currently investigating this further. The presence of misfolded α-synuclein prionoids in the spinal cord further suggests that after reaching the brain stem, possibly via the hypoglossal nerve, α-synuclein prionoids spread from the brain stem to the spinal cord. The underlying mechanism for how α-synuclein prionoids enter the CNS after injection into the peritoneal cavity is unclear. Possible mechanisms of neuroinvasion may include retrograde spread of infectivity along peripheral nerves or the hematogenous route, as discussed for prions (36). It has been hypothesized that in patients with PD, α-synuclein pathology commences in neurons of the olfactory bulb or in neurons of the enteric nervous system and then reaches the brain at the dorsal motor nucleus via the vagus nerve before it further spreads in the brain according to described staging patterns (37, 38). This hypothesis is supported by observations in rats, in which viral overexpression of native α-synuclein in the vagus nerve or injection of α-synuclein prionoids into the wall of the gastrointestinal tract led to retrograde transport of misfolded α-synuclein along the vagus nerve to the brain (39, 40). Retrograde transport along peripheral nerves was also discussed as a mode of neuroinvasion after intramuscular injection with 10 μg of fibrillary mouse α-synuclein into the gastrocnemius muscle in TgM83^+/−^ mice, which developed neurologic disease with a median incubation time of ∼129 days when the sciatic nerve was intact and showed delayed and incomplete induction of disease when the sciatic nerve was transected (41). Another study in rats recently showed that repeated intravenous injections of misfolded recombinant α-synuclein species, every 2 weeks over a period of 4 months, led to their accumulation in the brain and spinal cord, suggesting that pathogenic α-synuclein species can cross the blood-brain barrier (42).

In patients with synucleinopathies, α-synuclein prionoids are not exclusive to the CNS but can also affect the peripheral nervous system innervating tissues and organs in the premotor phase, long before the disease is diagnosed (37, 43–47). Whether α-synuclein prionoids can accumulate and replicate in cells, tissues, and organs outside the nervous system, similarly to prions in follicular dendritic cells (FDC) and secondary lymphoid tissues prior to neuroinvasion, needs to be determined (48–50). Remarkably, red blood cells contain relatively large concentrations of α-synuclein and may potentially serve as a reservoir for the replication and dissemination of α-synuclein prionoids throughout the body (51). While accidental transmissions of synucleinopathies by blood transfusions or organ transplants from donors with subclinical disease have not been reported and seem unlikely, detection of such incidences could prove difficult considering that incubation times after peripheral transmission can span several decades for prion diseases, and possibly also for Alzheimer's disease (27, 52, 53). In summary, our results demonstrate that within the animal model of our choice, Tg(M83^+/−^:*Gfap*-luc^+/−^) mice overexpressing mutant human α-synuclein and firefly luciferase, α-synuclein prionoids hold neuroinvasive properties that lead to neuropathology and disease when α-synuclein is administered intraperitoneally or intraglossally.

## Supplementary Material

Supplemental material
