# Supplementary material for: Neuroinvasion of α-Synuclein Prionoids after Intraperitoneal and Intraglossal Inoculation
Source: J Virol. 2016 Sep 29;90(20):9182–93. doi: 10.1128/JVI.01399-16 (PMC5044858; doi:10.1128/JVI.01399-16)
Supplement: Supplemental material [file supp_90_20_9182__index.html]

Neuroinvasion of α-Synuclein Prionoids after Intraperitoneal and Intraglossal Inoculation — Supplemental material 

# Neuroinvasion of α-Synuclein Prionoids after Intraperitoneal and Intraglossal Inoculation

## Supplemental material

- Supplemental file 1 -

  Movie S1 (Intraperitoneal challenge with α-synuclein fibrils causes neurologic disease in bigenic Tg(M83+/–:*Gfap*-luc+/–) mice.)

  MOV, 3.0M
- Supplemental file 2 -

  Supplemental Movie Legend.

  PDF, 83K
