# Supplementary material for: Neuroinvasion of α-Synuclein Prionoids after Intraperitoneal and Intraglossal Inoculation
Source: J Virol. 2016 Sep 29;90(20):9182–93. doi: 10.1128/JVI.01399-16 (PMC5044858; doi:10.1128/JVI.01399-16)
Supplement: Supplemental material [file JVI.01399-16_zjv999181999so2.pdf]

Supplemental material

Movie S1 Intraperitoneal challenge with α-synuclein fibrils causes neurologic disease in bigenic Tg(M83$^{+/-}$:*Gfap*-luc$^{+/-}$) mice. In contrast to Tg(M83$^{+/-}$:*Gfap*-luc$^{+/-}$) mice that were injected with PBS and remained healthy throughout the course of the experiment, four out of five mice intraperitoneally challenged with α-synuclein fibrils lost weight, had a ruffled coat, and developed neurologic signs of disease that included tail rigidity, kyphosis, ataxia, and paralysis.
